# A Nonlinear MEMS Inertial Switch Fabricated by Induction-Electrode Through-Mask Electrochemical Micromachining

**DOI:** 10.3390/mi17091007

**Published:** 2026-08-26

**Authors:** Bingze Shang, Meng Li, Xiaochen Yang, Bingnan Liu, Huifeng Qiu, Yan Cui, Liqun Du

**Affiliations:** 1 Key Laboratory for Micro/Nano Technology and System of Liaoning Province, Dalian University of Technology, Dalian 116024, China; qq1350496295@163.com (B.S.); 18339165839@163.com (M.L.); 18940886321@163.com (X.Y.); lbn2982174265@163.com (B.L.); hf_q519@163.com (H.Q.); yanc@dlut.edu.cn (Y.C.); 2 State Key Laboratory of High-Performance Precision Manufacturing, Dalian University of Technology, Dalian 116024, China

**Keywords:** MEMS inertial switch, geometric nonlinearity, threshold accuracy, induction-electrode through-mask electrochemical micromachining

## Abstract

To improve the threshold accuracy of inertial switches, this study proposes a monolithic metal MEMS inertial switch with nonlinear springs. The switch uses two sets of inclined beams with asymmetric initial angles as suspension springs. Geometric nonlinearity produces low displacement sensitivity away from the design threshold and high sensitivity near the threshold. This response improves threshold discrimination and reduces the deviation between the actual and design thresholds. A nonlinear switch and a linear reference switch are designed with the same static threshold of 27.5 g. Their responses are compared using Abaqus static and explicit dynamic simulations. Both switches are monolithically fabricated from 50 μm thick 304 stainless steel by induction-electrode through-mask electrochemical micromachining (IETMEMM). Key dimensional deviations are below 2.5%. Drop-weight tests show measured nonlinear-switch thresholds of 27.8, 27.8, 26.9, and 25.4 g under half-sine shocks with pulse widths of 4, 6, 8, and 10 ms, respectively. The maximum threshold deviation is 2.1 g. The overall threshold accuracy is 92.4%, substantially higher than the 56.0% of the linear reference switch. This work combines a nonlinear threshold-regulation mechanism with monolithic IETMEMM fabrication and provides a new strategy for metal MEMS inertial switches with high threshold accuracy.

## 1. Introduction

MEMS inertial switches offer several advantages, including a small size, low standby power consumption, a fast response, and direct threshold determination [1,2]. They have promising applications in automotive safety [3], transportation monitoring [4], and fall detection [5]. For a qualitative comparison, this study estimates the threshold accuracy of existing devices from the deviation between the reported triggering threshold and the nominal design value [6]. Because the test conditions differ among studies, these values are used only to characterize the degree of threshold deviation and are not intended as strictly equivalent performance metrics. Nevertheless, the reported data indicate that substantial deviations between measured and design thresholds remain common in MEMS inertial switches. For example, Fathalilou et al. designed a switch with a threshold of 200 g, whereas its minimum measured triggering threshold was only 115 g. Its threshold accuracy was 57.5% [7]. Xu et al. designed a switch with a threshold of 240 g. However, its measured threshold varied with the test conditions and reached 340 g. Its threshold accuracy was only 58.3% [8]. Low threshold accuracy reduces the reliability of detection results and severely limits the large-scale application of MEMS inertial switches.

Several methods have been proposed to reduce the deviation between the measured triggering threshold and the design value and thereby improve threshold accuracy. Zhang et al. [9] used a symmetric double-buried-oxide SOI structure to independently optimize a large proof mass and low-stiffness springs. This method limited the maximum measured threshold deviation of a device with a design threshold of 5.5 g to less than 0.73 g. The resulting threshold accuracy was approximately 86.7%. Peng et al. [10] improved threshold accuracy through passive dynamic regulation. They used the compensating relationship between squeeze-film damping and the elastic restoring force. This approach limited the absolute measured threshold deviation of a 5 g device to less than 0.8 g and achieved a threshold accuracy of 84%. These two methods improve threshold accuracy to some extent. However, the low fracture toughness of silicon limits the overload resistance of these devices [11]. To improve both threshold accuracy and overload resistance, Du et al. [12] developed a metal inertial switch. They compensated for thickness variations by adjusting the structural dimensions. The omnidirectional inertial switch had a nominal threshold of 38 g and measured thresholds of 35–40 g, corresponding to a threshold accuracy of 92.1%. However, this method requires the thickness of a subsequent compensation layer to be adjusted according to the result of the preceding fabrication step, which increases process complexity [13].

The methods described above improve the threshold accuracy of inertial switches to some extent. However, all these switches use linear structures. Their responses to loads near the design threshold are insufficiently distinguishable, which can cause large threshold deviations [14]. Nonlinear structures can improve response discrimination near a target load [15]. However, metal MEMS inertial switches that use nonlinear structures to improve threshold accuracy have not yet been reported. Therefore, this study proposes a nonlinear metal MEMS inertial switch. The switch uses geometric nonlinearity to better distinguish between shocks below and above the design threshold. This behavior reduces the deviation between the dynamic triggering threshold and the design threshold and improves threshold accuracy.

## 2. Operating Principle and Structural Design of the Nonlinear Inertial Switch

### 2.1. Operating Principle

**Figure 1:**
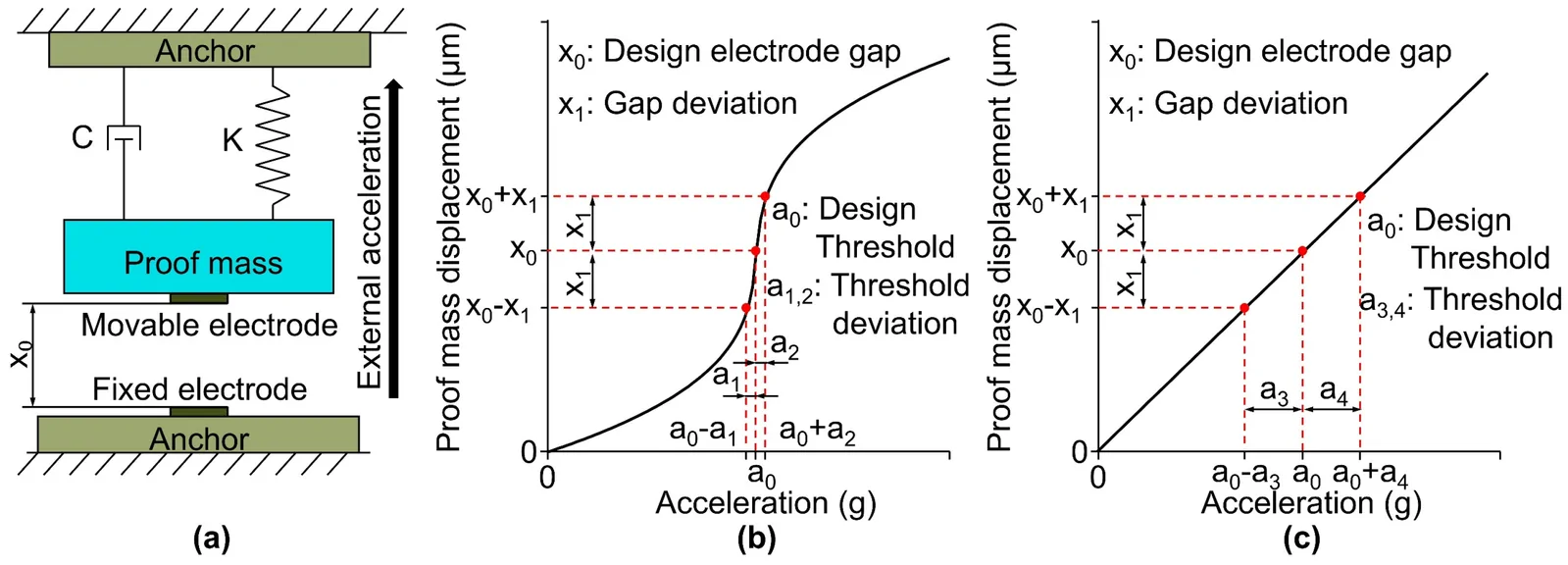
Operating principle of a conventional MEMS inertial switch and comparison of the sensitivities of linear and nonlinear displacement–acceleration responses to electrode-gap deviations. (**a**) Equivalent single-degree-of-freedom mass–spring–damper model of the inertial switch. (**b**) Proof–mass displacement–acceleration response of the nonlinear structure. (**c**) Proof-mass displacement–acceleration response of the linear structure.

The dynamic behavior of a conventional MEMS inertial switch can generally be represented by a single-degree-of-freedom mass–spring–damper system. Its basic structure consists of elastic suspension beams, a proof mass, a fixed electrode, and anchor regions. The proof mass usually also serves as the movable electrode [16,17]. As shown in Figure 1a, one end of each suspension beam is fixed to an anchor region, while the other end is connected to the suspended proof mass. When the device is subjected to a half-sine acceleration shock along the sensing direction, the proof mass moves relative to the substrate under the inertial force. As the relative displacement increases, the movable electrode contacts the fixed electrode when the proof-mass displacement reaches the initial electrode gap $x_{0}$. The switch then closes and forms a conductive path, thereby generating an electrical trigger signal [17,18]. For a given shock waveform, pulse width, and test condition, the minimum peak shock acceleration that just closes the switch is defined as the threshold acceleration $a_{th}$ [17,18]. The displacement response of the proof mass is mainly governed by the stiffness K of the suspension structure. Therefore, the stiffness is a key parameter that determines the displacement–acceleration response and triggering threshold of the inertial switch.

The stiffness of a conventional linear inertial switch is approximately constant. Therefore, the proof-mass displacement varies almost linearly with the applied acceleration. In this study, geometric nonlinearity is introduced to make the stiffness vary with displacement. This behavior produces a nonlinear displacement–acceleration response. As shown in Figure 1b,c, the acceleration $a_{0}$, at which the proof-mass displacement reaches the designed electrode gap $x_{0}$, is defined as the design threshold for both the linear and nonlinear structures. However, the measured electrode gap inevitably deviates from its design value because of fabrication errors, assembly errors, and residual stress. The electrode-gap deviation shifts the triggering threshold and reduces threshold accuracy. When the actual electrode gaps become $x_{0}-x_{1}$ and $x_{0}+x_{1}$, the corresponding thresholds of the nonlinear structure become $a_{0}-a_{1}$ and $a_{0}+a_{2}$, respectively, where $a_{1}\neq a_{2}$. For the linear structure, the corresponding thresholds become $a_{0}-a_{3}$ and $a_{0}+a_{4}$, where $a_{3}=a_{4}$. For the same gap deviation $x_{1}$, $a_{1}<a_{3}$ and $a_{2}<a_{4}$. This result indicates that the nonlinear structure has higher displacement sensitivity near the design operating point. Therefore, when $a_{0}$ is used as the design threshold, the proposed nonlinear structure reduces the threshold drift caused by electrode-gap deviations. It thereby improves threshold accuracy and helps improve device consistency.

Threshold accuracy is a dimensionless metric that measures the agreement between the actual triggering threshold and the design threshold of an inertial switch. It reflects the ability of the device to maintain its design threshold [10]. The measured threshold of an inertial switch is affected by pulse width, acceleration waveform, structural damping, and fabrication errors. Therefore, the absolute threshold deviation alone does not allow a direct comparison between different test conditions or devices with different design thresholds. In this study, the static design threshold is used as the reference value. The absolute deviation between the measured threshold and the design threshold is normalized. Threshold accuracy is then defined as the complement of the relative deviation [6]. This metric expresses the threshold deviation as a percentage. A higher threshold accuracy indicates that the actual triggering threshold is closer to the design value and that the device has a stronger threshold-retention capability. Furthermore, the maximum relative deviation among all test conditions is used to identify the worst-case condition. The overall threshold accuracy is calculated from this condition to evaluate the overall threshold-retention performance of the device.

The design thresholds of existing MEMS inertial switches are generally determined under quasi-static conditions. However, the shock load under actual operating conditions can be approximated by a half-sine acceleration pulse with a specific peak acceleration and pulse width. Even at the same peak acceleration, a change in pulse width can produce a different maximum proof-mass displacement. This difference can change the dynamic triggering threshold of the switch. Therefore, the triggering threshold under quasi-static conditions is used as the design threshold in this study. The dynamic triggering thresholds under different pulse widths are then analyzed to evaluate the threshold accuracy of the switch.

First, the relative threshold deviation under the ith test condition is defined as [6]:

(1) $$\varepsilon_{i}=\frac{\left|a_{\mathrm{dyn},i}-a_{0}\right|}{a_{0}}\times 100\%$$

Based on the relative deviation defined above, the threshold accuracy under the ith test condition is defined as its complement [6]:

(2) $$\delta_{i}=\left(1-\frac{\left|a_{\mathrm{dyn},i}-a_{0}\right|}{a_{0}}\right)\times 100\%=100\%-\varepsilon_{i}$$

Here, $a_{0}$ is the quasi-static design threshold, and $a_{0}=27.5\;g$ in this study. $a_{dyn,i}$ is the dynamic triggering threshold under the ith pulse-width condition. $\varepsilon_{i}$ is the relative threshold deviation at the corresponding pulse width, and $\delta_{i}$ is the corresponding threshold accuracy. According to Equations (1) and (2), a dynamic triggering threshold closer to the design threshold produces a smaller relative deviation and a higher threshold accuracy. To evaluate the overall threshold-retention capability of the device under different test conditions, the maximum relative deviation among all conditions is used as the evaluation criterion. The corresponding test condition is defined as the worst-case condition. The overall threshold accuracy is defined as [6]:

(3) $$\delta_{\mathrm{overall}}=100\%-{max}_{i}\left(\varepsilon_{i}\right)$$

This definition reflects the extent of threshold deviation under the worst-case test condition and thus provides a more stringent evaluation of the overall threshold-retention performance of the device.

### 2.2. Structural Design

**Figure 2:**
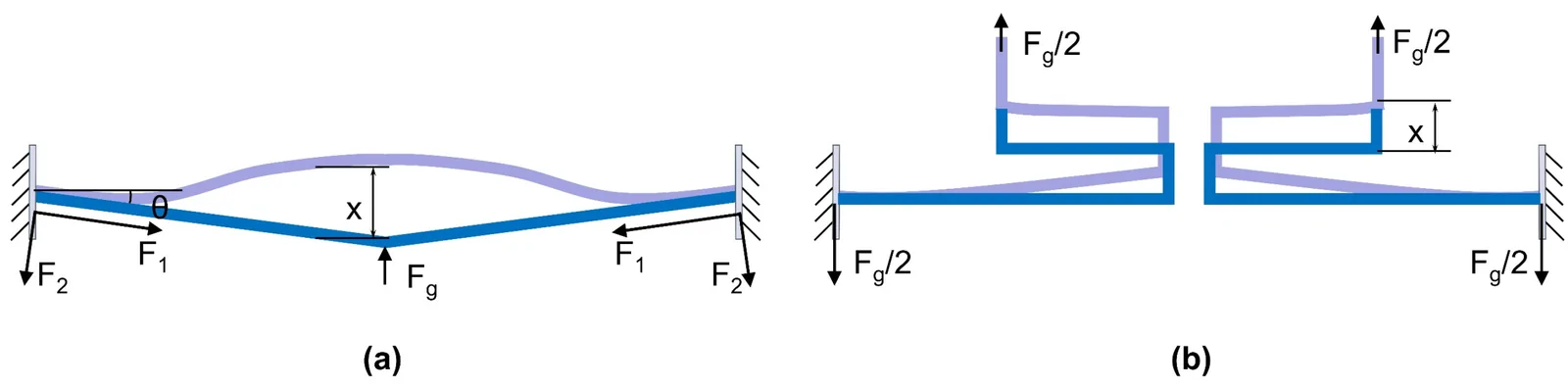
Deformation and force analysis of the suspension beams. The blue lines represent the undeformed configurations, and the purple lines represent the deformed configurations. (**a**) Inclined beam. (**b**) Serpentine beam.

To achieve high threshold accuracy through a nonlinear acceleration–displacement response, inclined beams are introduced in this study. Unlike serpentine beams with linear characteristics, the inclined beams undergo not only bending but also axial compression under the external force $F_{g}$, as shown in Figure 2. The axial compressive force $F_{1}$ is the fundamental source of the nonlinear behavior [19]. Both the initial inclination angle θ of the beam and the displacement x at point P affect the amount of axial compression and, consequently, the magnitude of the axial force $F_{1}$. Therefore, both θ and δ are important design parameters of the inclined beam. To quantitatively investigate their effects on the nonlinear behavior, the mechanical characteristics of the inclined beam are analyzed through finite element simulations in Section 3.1.

**Figure 3:**
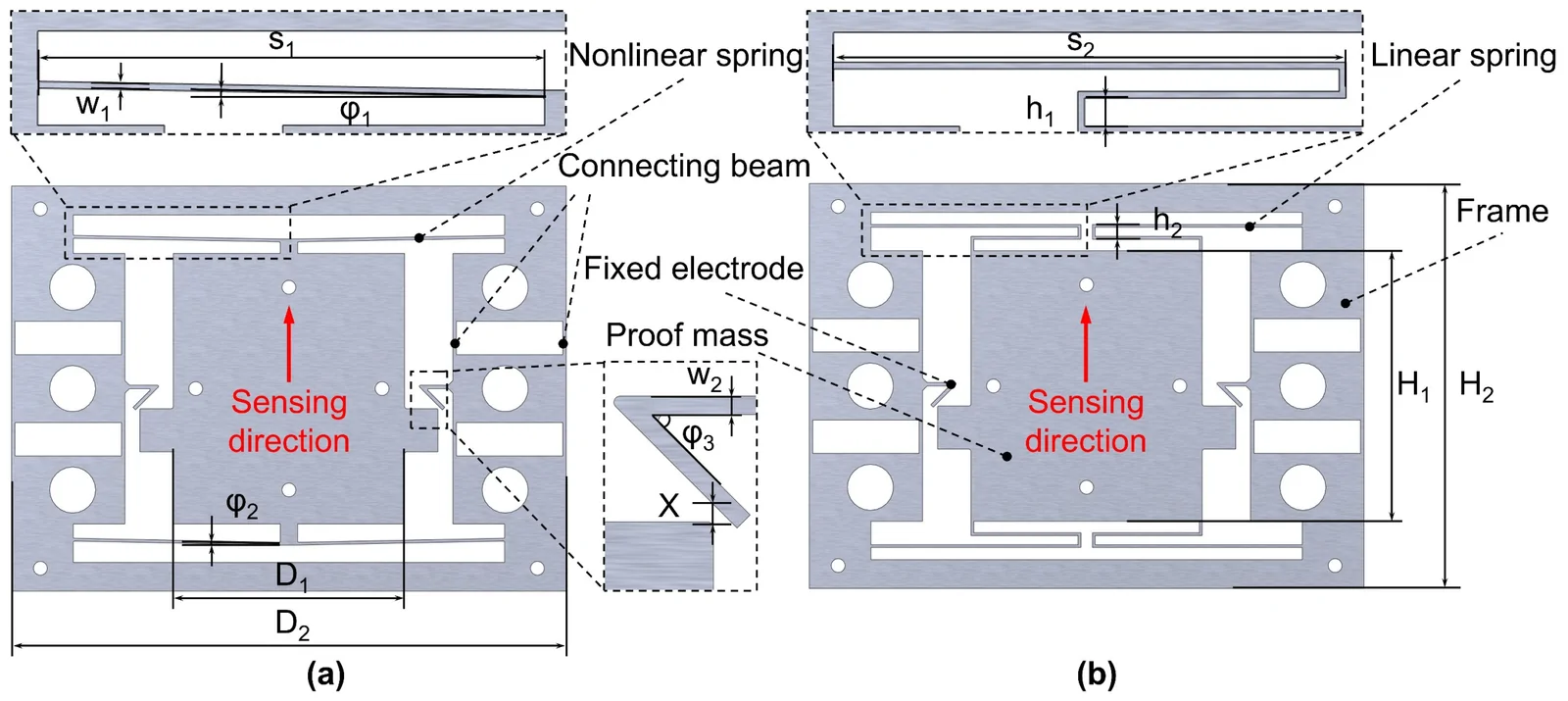
Structure and key geometric parameters of the inertial switches. The device thickness is t = 50 μm, perpendicular to the device plane. (**a**) Nonlinear inertial switch. (**b**) Linear inertial switch.

The structure of the nonlinear inertial switch is shown in Figure 3a. It mainly consists of nonlinear suspension springs, a proof mass, fixed electrodes, and an outer frame. The proof mass serves as the inertial sensing element, with its upper and lower ends connected to two sets of suspension springs and movable contacts arranged on both sides. The outer frame supports the device, protects the chip, and facilitates subsequent PCB assembly. The nonlinear suspension springs are key to regulating the dynamic displacement response of the proof mass and suppressing threshold drift. Each spring consists of two sets of inclined beams with asymmetric initial angles, resulting in displacement-dependent stiffness during loading. To improve contact stability during dynamic closure, flexible fixed electrodes are arranged on the outer frame, since shock loading may cause contact bounce or insufficient contact duration, leading to transient contact or unstable output signals [17,20]. After assembly, the connecting beams are removed to separate the fixed electrodes from the frame as independent conductive units. Their local deformation absorbs contact impact and improves dynamic contact stability without introducing additional complex mechanisms around the proof mass, thereby maintaining structural simplicity and monolithic fabrication feasibility.

To evaluate the contribution of the nonlinear suspension structure to threshold accuracy, a linear MEMS inertial switch is designed as the reference device, as shown in Figure 3b. The linear switch uses symmetric serpentine beams [21] as suspension springs instead of nonlinear inclined beams. Except for the suspension-spring configuration, the proof mass, fixed electrodes, outer frame, and electrode gap are identical in the two devices. This design ensures that differences in their dynamic triggering behavior mainly arise from the suspension structures.

The key geometric parameters of the two inertial switches are listed in Table 1. The device thickness is 50 μm, and the electrode gap is 180 μm. The spring-beam width is 120 μm for both switches. For the nonlinear switch, the stiffness and displacement response are regulated by adjusting the beam length $S_{1}$ and the initial angles $\varphi_{1}$ and $\varphi_{2}$ of the upper and lower inclined beams. For the linear switch, the serpentine-beam length $S_{2}$ is adjusted so that its static triggering threshold is the same as that of the nonlinear switch. This design ensures a valid comparison between the two structures.

## 3. Simulation

Abaqus is used to perform quasi-static and transient dynamic analyses of the MEMS inertial switches. The quasi-static analysis determines the design threshold and the displacement–acceleration relationship. The transient dynamic analysis evaluates the modal characteristics of the dynamic response and the dynamic triggering behavior under different shock acceleration amplitudes and pulse widths.

### 3.1. Static Simulation Analysis

**Figure 4:**
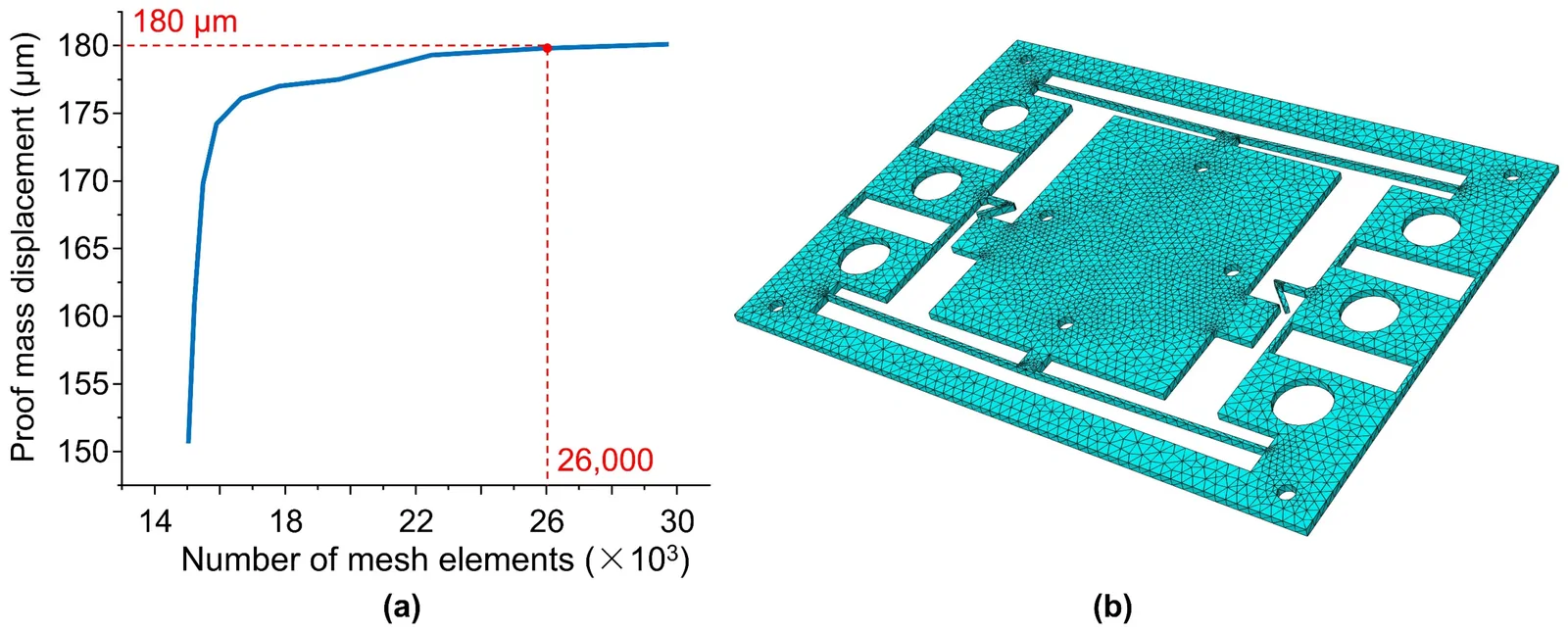
Mesh independence study. (**a**) Effect of the number of mesh elements on the proof–mass displacement. (**b**) Final mesh configuration with 26,000 elements.

To determine the static triggering threshold and analyze the displacement–acceleration responses of the two structures under quasi-static loading, a static analysis is used to simulate the quasi-static loading process. The device material is 304 stainless steel. Its main material properties are a Young’s modulus of E=193 GPa, a density of ρ=7930 kg/m^3^, and a Poisson’s ratio of ν=0.29. In the static simulation, geometric nonlinearity was enabled, and fixed boundary conditions were applied to the external frame. The finite element models are meshed with tetrahedral elements. To balance computational accuracy and efficiency, a mesh-density convergence study is performed. As shown in Figure 4a, when the total number of mesh elements exceeds 26,000, the variation in proof-mass displacement becomes very small. Compared with the displacement obtained using a mesh with 30,000 elements, the difference is only 2%. Therefore, a mesh with 26,000 elements is adopted, as shown in Figure 4b.

**Figure 5:**
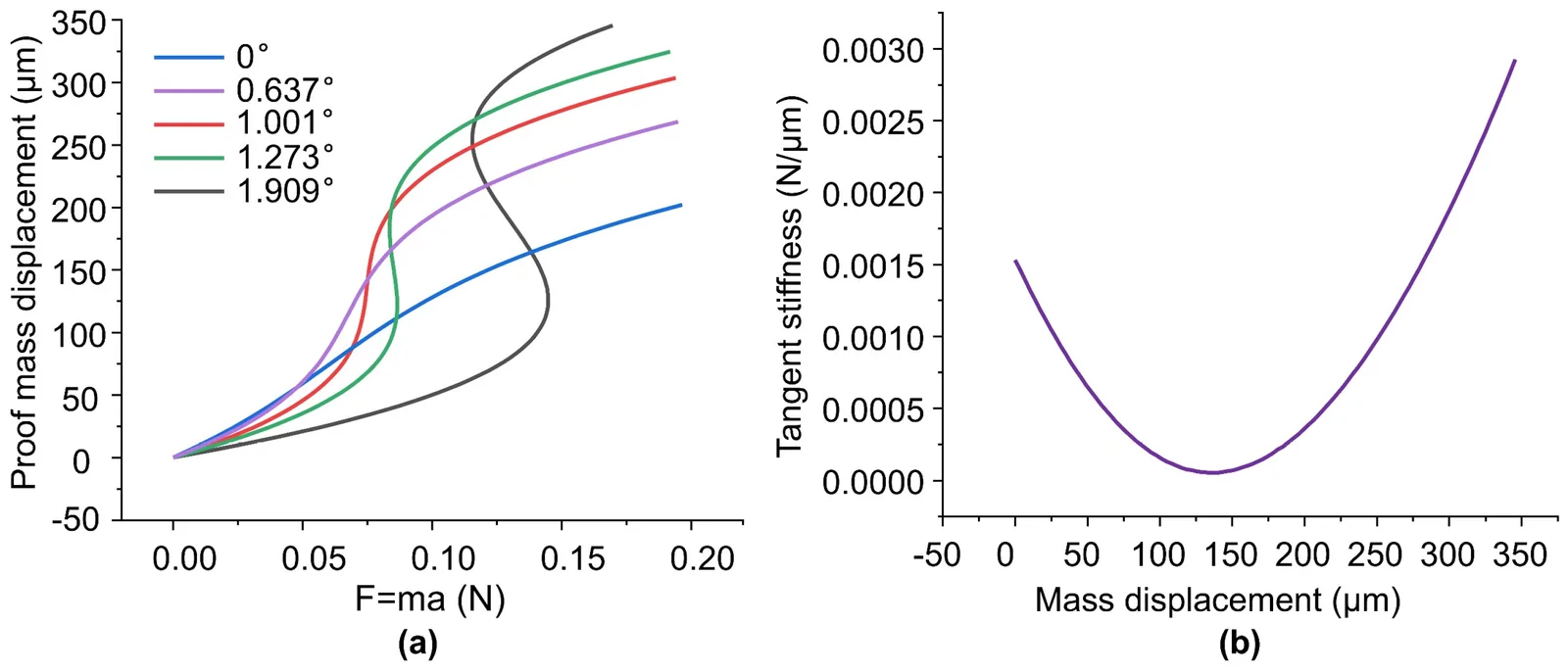
Angle–dependent displacement response and displacement–dependent tangent stiffness of the proof mass. (**a**) Force–displacement responses of the proof mass at different angles (0°, 0.637°, 1.001°, 1.273°, and 1.909°). (**b**) Tangent stiffness as a function of proof–mass displacement derived from the force-displacement response at 1.001° (red curve in panel (**a**)).

According to Section 2.2, the inclination angle is a key geometric parameter governing the nonlinear behavior; therefore, a parametric optimization of the inclination angle is conducted. In the static simulation, the inclination angle of the lower beam is fixed while that of the upper beam is varied, and the results are shown in Figure 5a. When the inclination angle is small, the force–displacement response shows weak nonlinearity. Therefore, its effect on the displacement response and threshold discrimination is limited. When the inclination angle is too large, a negative-stiffness region appears, which makes the structural response difficult to control [19,22]. At an inclination angle of 1.001°, the force–displacement curve shows clear nonlinear behavior and meets the design requirements. Therefore, 1.001° and 1.146° are selected as the design angles of the upper and lower beams, respectively.

After the optimal parameters are determined, the data for the 1.001° case are extracted to obtain the tangent stiffness curve, as shown in Figure 5b. The tangent stiffness first decreases and then increases with displacement. This trend is consistent with the designed nonlinear spring behavior, in which the stiffness decreases first and then increases as the force and displacement increase.

**Figure 6:**
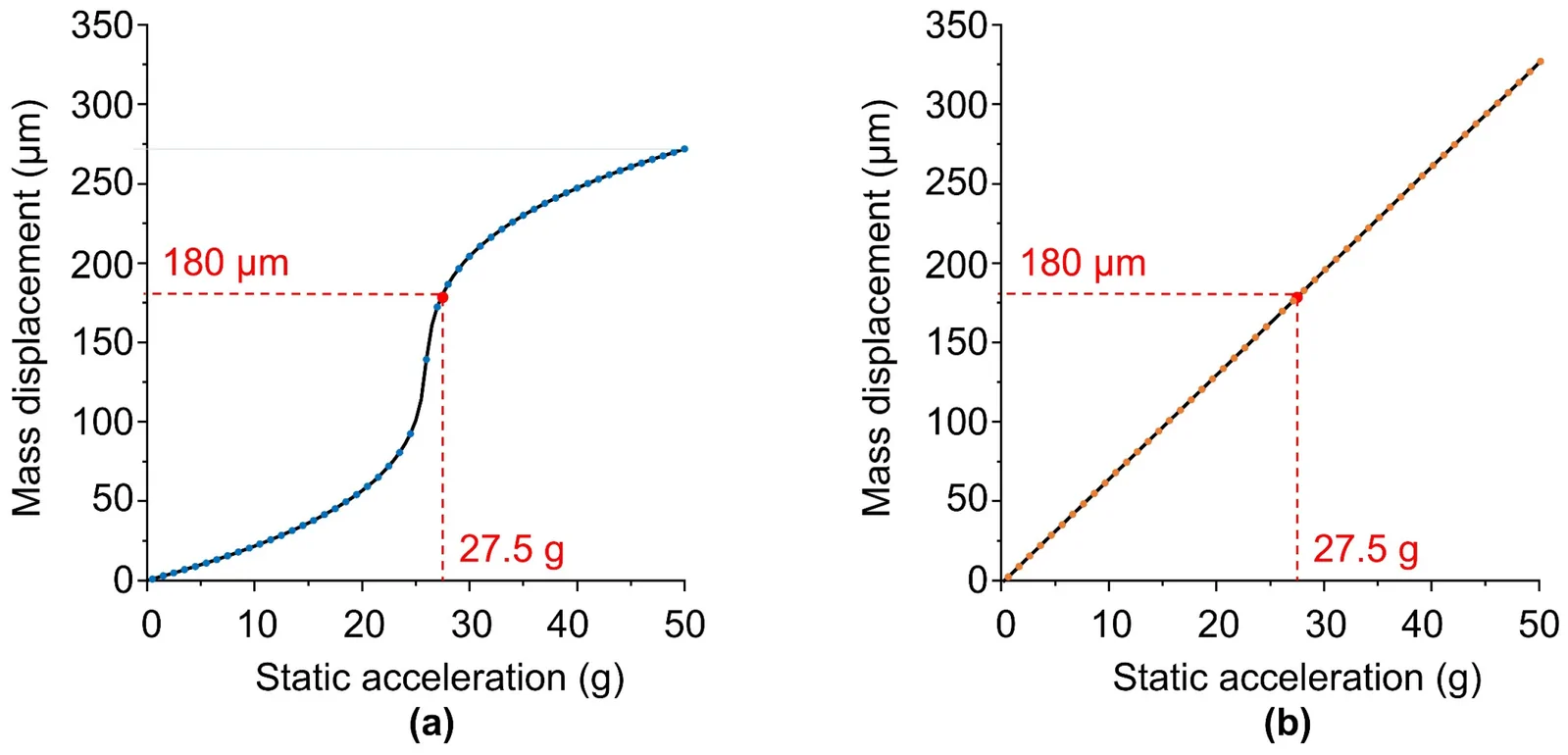
Displacement of the proof mass under static acceleration. (**a**) Proof–mass displacement–acceleration response of the nonlinear inertial switch under static acceleration. (**b**) Proof–mass displacement–acceleration response of the linear inertial switch under static acceleration.

Equivalent acceleration loads are gradually increased from 0 to 50 g for both inertial switches to simulate their quasi-static responses at different acceleration levels. The proof-mass displacement along the sensing direction is then extracted. The resulting displacement–acceleration relationships are shown in Figure 6. A total of 100 data points are obtained from the simulations. To improve the readability of the curves, 50 data points are selected at equal intervals for plotting. The static acceleration increment between adjacent displayed points is 1 g. The discrete markers represent the selected simulation results, while the solid lines indicate the overall trends of the data.

As shown in Figure 6a, the mass displacement of the nonlinear inertial switch increases nonlinearly with acceleration. This result indicates that the stiffness is not constant during loading. Instead, it varies with the mass displacement and load level, which agrees with the trend shown in Figure 1b. When the acceleration is below 22.5 g, the proof-mass displacement increases slowly. This behavior indicates that the nonlinear suspension springs reduce displacement sensitivity in the low-load range. When the acceleration is between 22.5 and 30 g, the slope of the displacement–acceleration curve increases markedly. In this range, a small acceleration increment produces a large displacement increment. Therefore, the proof mass can reach the electrode gap with only a small increase in acceleration near the design threshold. At an acceleration of 27.5 g, the proof-mass displacement reaches 180 μm, which is equal to the designed electrode gap and represents the design triggering displacement. Therefore, the quasi-static triggering threshold of the nonlinear inertial switch is 27.5 g, and this value is used as the design threshold.

In contrast, the linear switch shown in Figure 6b does not exhibit a localized increase in displacement sensitivity near the design threshold. Its displacement–acceleration relationship remains approximately linear over the analyzed range. This behavior is consistent with a linear elastic system with nearly constant stiffness. During structural design, the geometric parameters and stiffness of the serpentine beams are adjusted so that the linear inertial switch also produces a proof-mass displacement of 180 μm at 27.5 g. Therefore, the quasi-static triggering threshold of the linear inertial switch is also 27.5 g.

These results verify the theoretical analysis presented in Section 2.1. Compared with the linear switch, the nonlinear switch concentrates high displacement sensitivity near the design threshold. A small change in acceleration therefore produces a large change in displacement in this region. This response helps confine the actual transition to the triggered state near the design threshold, thereby improving the threshold accuracy of the inertial switch.

### 3.2. Dynamic Simulation Analysis

**Figure 7:**
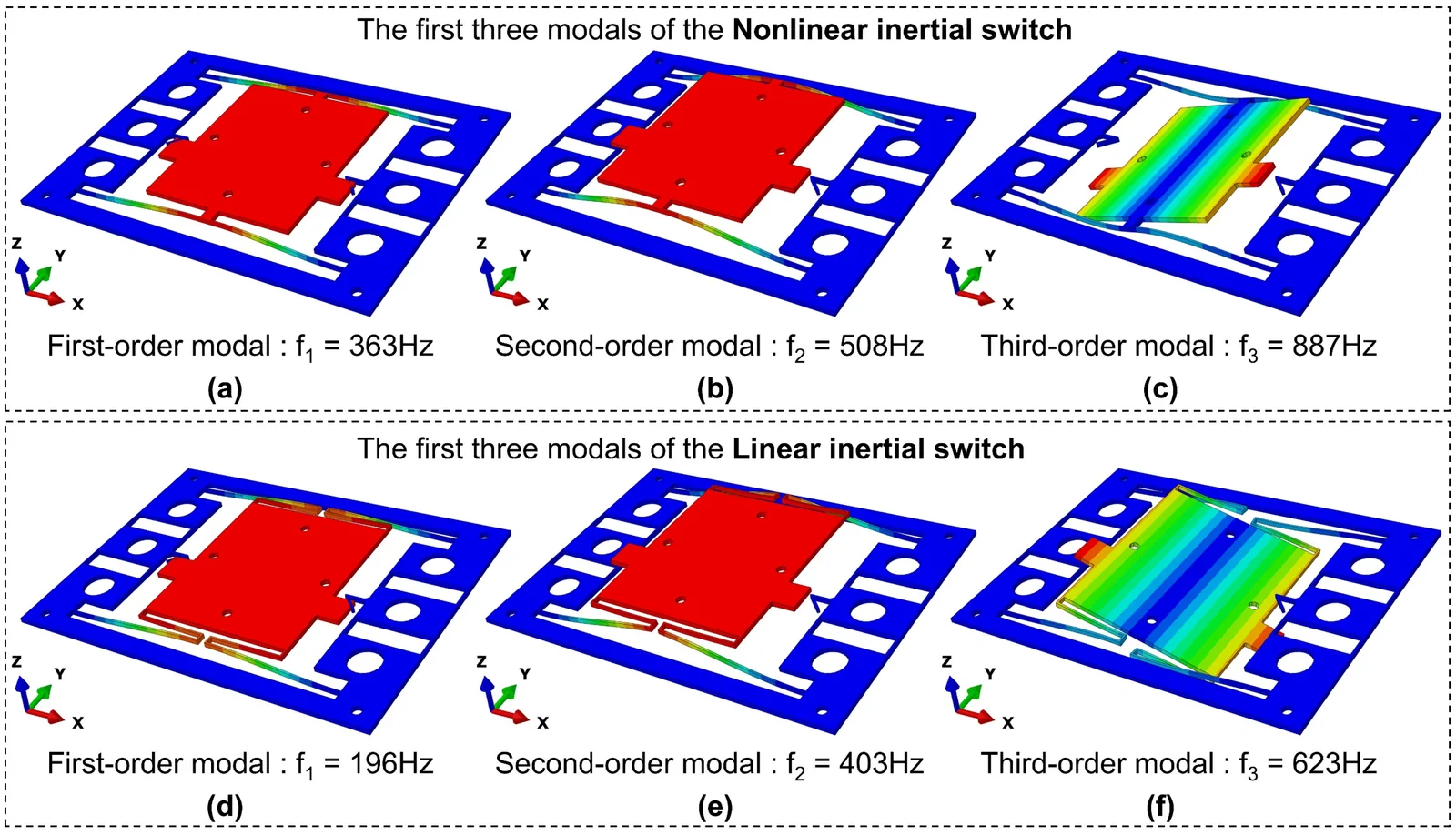
Modal analysis results of the nonlinear and linear inertial switches. The color contour represents the relative displacement, with blue indicating zero displacement and colors approaching red indicating progressively larger displacement from the original position. (**a**–**c**) First three modes of the nonlinear inertial switch. (**d**–**f**) First three modes of the linear inertial switch.

To further evaluate the dynamic responses of the inertial switches, transient dynamic simulations are performed using the same structural parameters, material properties, and boundary conditions as those adopted in the static simulations. The first three vibration modes of the nonlinear and linear switches are shown in Figure 7. As presented in Figure 7a,d, the first natural frequencies associated with motion in the Y direction are 363 Hz for the nonlinear switch and 196 Hz for the linear switch, respectively. The higher first natural frequency of the nonlinear switch indicates that the nonlinear suspension structure provides a higher effective dynamic stiffness around the initial equilibrium position. A higher natural frequency also reduces the susceptibility of the proof mass to low-frequency environmental disturbances and structural vibrations, thereby limiting unintended displacement before the prescribed acceleration threshold is reached. This characteristic is beneficial for improving the dynamic stability of the inertial switch and reducing the possibility of false triggering under external disturbances. In addition, the increased dynamic stiffness of the nonlinear suspension is expected to influence the transient displacement evolution of the proof mass under shock loading, which is further examined in the following transient dynamic analysis.

Transient dynamic simulations are then performed using an explicit dynamic solver with geometric nonlinearity enabled. The total simulation time is set to 10 ms, with a maximum time increment of $10^{-5}$ s. No contact constraints are applied to characterize the displacement evolution of the proof mass. Both switches operate with in-plane motion, and their damping is mainly slide-film damping. Therefore, damping is neglected in the simulations [10]. For different simulation conditions, half-sine acceleration pulses with different peak accelerations and pulse widths are applied as input excitations. Y-direction accelerations with different amplitudes are applied, and the corresponding half-sine amplitude curves are defined for different pulse widths. The transient proof-mass displacement under shock loading is then extracted. The initial gap between the movable and fixed electrodes is 180 μm. Therefore, the inertial switch is considered closed when the maximum proof-mass displacement reaches 180 μm. Half-sine acceleration pulses with pulse widths of 4, 6, 8, and 10 ms are selected to evaluate threshold stability under pulse-width variations and to provide a basis for analyzing resistance to false triggering. This study focuses on the switch response over a pulse-width range of 4–10 ms. In addition, to verify whether the switch response at a pulse width of 10 ms can be reasonably approximated as quasi-static, an additional pulse width of 14 ms was introduced solely as an extended validation condition. The results at 14 ms were not included in the threshold analysis or statistical evaluation.

**Figure 8:**
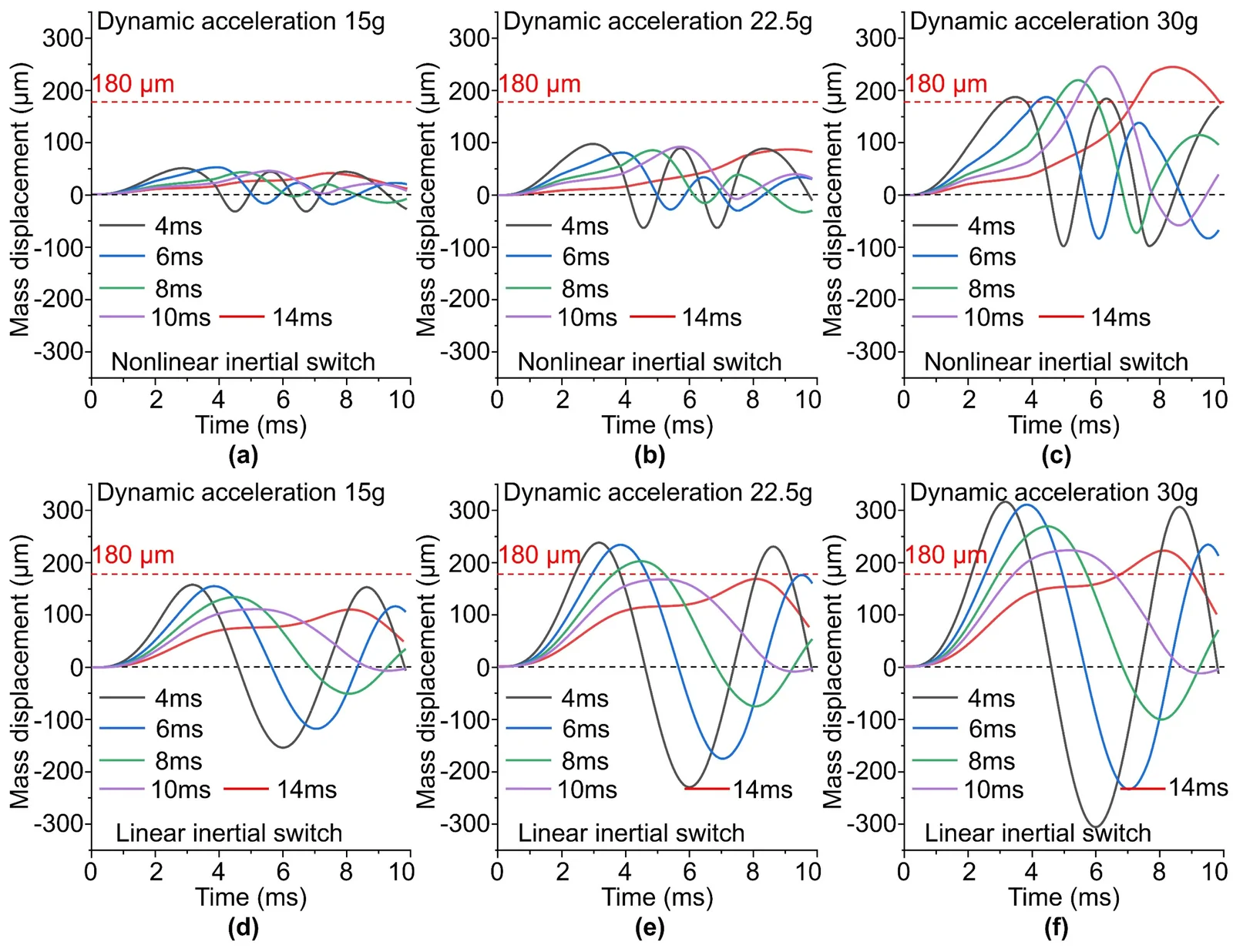
Transient proof–mass displacement under half–sine acceleration pulses with widths of 4, 6, 8, 10, and 14 ms. (**a**–**c**) Proof–mass displacement–acceleration response of the nonlinear inertial switch under dynamic accelerations of 15, 22.5, and 30 g. (**d**–**f**) Proof–mass displacement–acceleration response of the linear inertial switch under dynamic accelerations of 15, 22.5, and 30 g.

The dynamic displacement responses of the nonlinear inertial switch are shown in Figure 8a–c. At input accelerations of 15 and 22.5 g, the maximum proof-mass displacement remains below 180 μm for all pulse widths from 4 to 10 ms. Therefore, the switch remains open. When the input acceleration increases to 30 g, the maximum proof-mass displacement exceeds 180 μm under all pulse-width conditions and satisfies the closure criterion. Based on these discrete acceleration levels, the dynamic triggering threshold of the nonlinear inertial switch is greater than 22.5 g and no higher than 30 g over the pulse-width range of 4–10 ms. This range contains the quasi-static design threshold of 27.5 g. The same threshold range is obtained for all tested pulse widths. This result indicates that pulse-width variation has a limited effect on the triggering boundary of the nonlinear switch within the tested pulse-width range and acceleration resolution. Therefore, the nonlinear stiffness design helps improve dynamic threshold stability.

The dynamic displacement responses of the linear inertial switch are shown in Figure 8d–f. Unlike the nonlinear switch, the proof-mass displacement of the linear switch increases approximately linearly with the input acceleration. Changes in pulse width clearly affect its maximum displacement and displacement–acceleration relationship. Therefore, the maximum displacement response of the linear structure varies more strongly when the shock pulse width changes. At an input acceleration of 15 g, the linear switch does not trigger under any pulse-width condition. At 22.5 g, the maximum proof-mass displacements for different pulse widths lie on both sides of the critical displacement of 180 μm. This result indicates that the switch closes under some conditions but remains open under others. When the input acceleration increases to 30 g, the switch closes under all pulse-width conditions. Based on these discrete loading cases, the dynamic triggering thresholds for different pulse widths are distributed over an overall range greater than 15 g and no higher than 30 g. Compared with the nonlinear inertial switch, the linear switch has a wider overall range of dynamic triggering thresholds. Some of these thresholds also show large deviations from the design threshold. These results indicate that the linear switch is more sensitive to changes in shock pulse width.

Based on the dynamic analysis above, the peak acceleration increment of the half-sine pulses is further reduced. The dynamic triggering thresholds of both switches under different pulse widths are then determined, as listed in Table 2. The quasi-static simulations reveal how the nonlinear structure improves threshold discrimination. The agreement between the dynamic triggering thresholds and the quasi-static design threshold quantifies the resulting improvement in threshold accuracy. This agreement is evaluated using the relative threshold deviation and threshold accuracy defined above. The threshold range across different pulse widths is also used to characterize threshold stability. A narrower range indicates lower sensitivity to pulse-width variation.

As shown in Table 2, the dynamic triggering thresholds of the nonlinear inertial switch are 29.5, 29.5, 28.4, and 26.9 g at pulse widths of 4, 6, 8, and 10 ms, respectively. The corresponding absolute deviations from the quasi-static design threshold of 27.5 g are 2.0, 2.0, 0.9, and 0.6 g. The thresholds range from 26.9 to 29.5 g, giving a span of only 2.6 g. According to Equations (1) and (2), the relative deviations are 7.3%, 7.3%, 3.3%, and 2.2%, and the corresponding threshold accuracies are 92.7%, 92.7%, 96.7%, and 97.8%. According to Equation (3), the worst-case overall threshold accuracy is 92.7%. These results indicate that the nonlinear structure limits shifts in the triggering boundary caused by pulse-width variation and keeps the dynamic thresholds close to the design value.

Under the same pulse-width conditions, the dynamic triggering thresholds of the linear inertial switch are 17.2, 17.4, 20.2, and 24.3 g, all below the design threshold. The corresponding absolute deviations are 10.3, 10.1, 7.3, and 3.2 g. The thresholds range from 17.2 to 24.3 g, with a span of 7.1 g. The relative deviations are 37.5%, 36.7%, 26.5%, and 11.6%, and the corresponding threshold accuracies are 62.5%, 63.3%, 73.5%, and 88.4%. The overall threshold accuracy is 62.5%.

Compared with the linear switch, the nonlinear switch reduces the maximum absolute deviation from 10.3 to 2.0 g, a reduction of 80.6%. The mean absolute deviation decreases from 7.73 to 1.38 g, a reduction of 82.2%. The maximum relative deviation decreases from 37.5% to 7.3%, while the overall threshold accuracy increases from 62.5% to 92.7%. These simulation results demonstrate that the nonlinear mechanism not only improves threshold discrimination under quasi-static conditions but also significantly enhances threshold accuracy and stability under dynamic shocks. In addition, the simulation results at a pulse width of 14 ms show that both the peak displacement and dynamic triggering threshold are close to those at 10 ms, indicating that the device response approaches the quasi-static response when the pulse width reaches 10 ms.

## 4. Fabrication and Testing

### 4.1. Fabrication

Nonlinear and linear MEMS inertial switches are fabricated by IETMEMM [23,24]. Unlike MEMS processes involving multilayer deposition, release, bonding, and assembly, IETMEMM forms the proof mass, suspension springs, fixed electrodes, and outer frame monolithically from a 50 μm-thick 304 stainless-steel sheet. This monolithic structure reduces assembly errors and interface-induced boundary variations. It also retains the good electrical conductivity, ductility, and impact resistance of the metal structure, providing a basis for batch fabrication.

**Figure 9:**
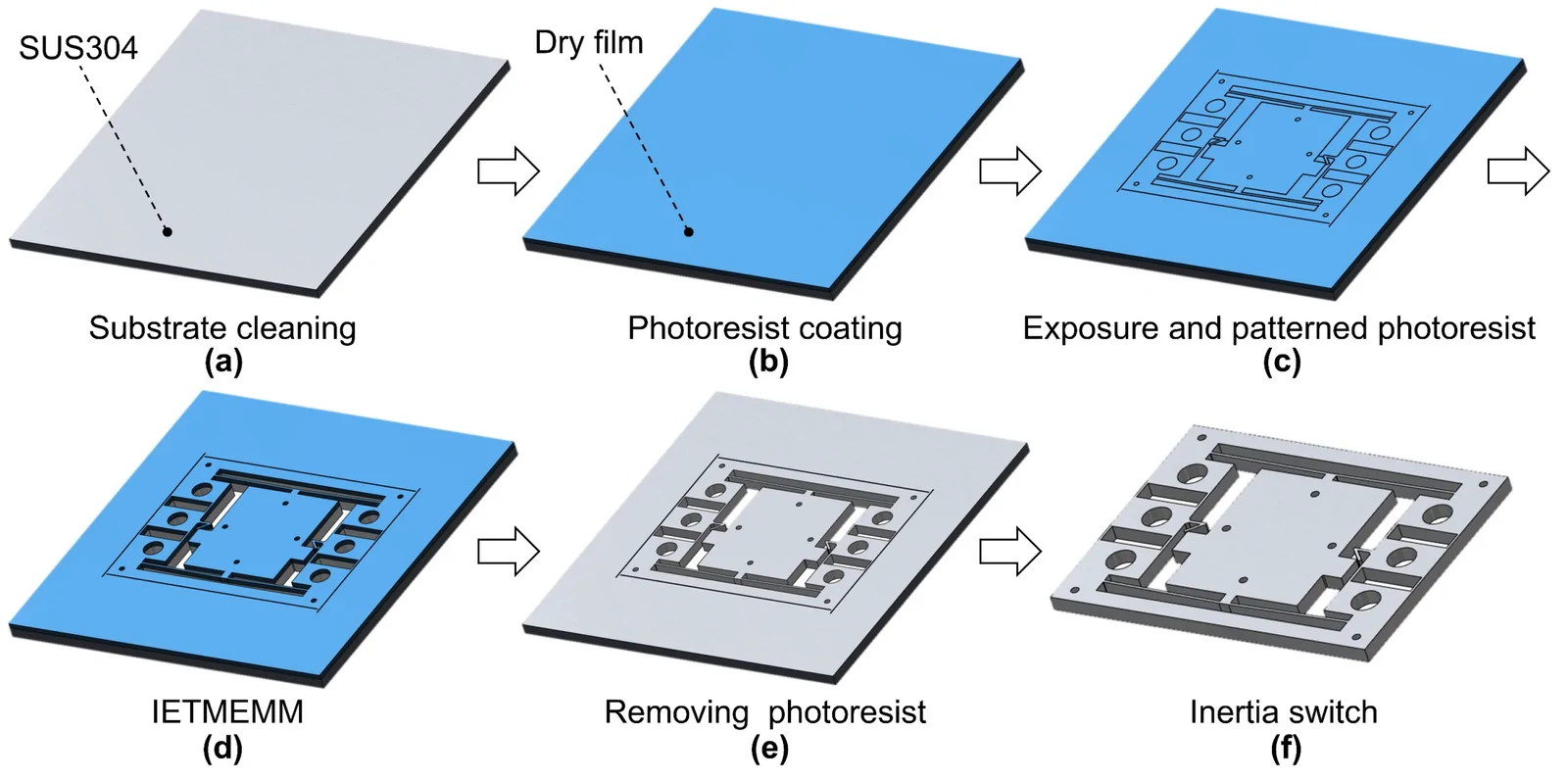
Fabrication process of the inertial switch. (**a**–**c**) Photoresist patterning. (**d**) Induction-electrode through-mask electrochemical micromachining (IETMEMM). (**e**) Photoresist removal and cleaning. (**f**) Completed inertial switch.

IETMEMM controls the electric-field distribution through a feeding electrode. The workpiece therefore acts as an induction electrode and undergoes electrochemical dissolution without direct connection to the power supply. Previous studies show that its self-stopping effect suppresses continuous lateral etching in through-hole structures and reduces the machining nonuniformity of MEMS inertial switches to 3.8%, providing higher accuracy than conventional through-mask electrochemical micromachining (TMEMM) [23]. The fabrication process is shown in Figure 9. The 304 stainless-steel sheet is first cleaned and pretreated. After wiping with acetone-soaked cotton, the sheet is ultrasonically cleaned in acetone and ethanol for 15 min each and then rinsed with deionized water. A photoresist is then laminated onto the sheet and patterned by exposure and development. A 15 μm-thick DuPont S200 photosensitive dry film is used. The exposure dose is 80 mJ, followed by development in a 0.86 wt.% Na_2_CO_3_ solution for 40 s.

The sample is then processed in the IETMEMM system using a 25 wt.% NaCl solution as the electrolyte. A two-stage machining strategy of 4 A for 20 s and 17 A for 8 s is adopted. According to previous studies, an excessively low current can cause anodic passivation and pitting, whereas an excessively high current can increase lateral corrosion and machining nonuniformity because of electrolyte-product accumulation and limited mass transfer. Therefore, a current density of 7 A/cm^2^ is selected as the baseline value to balance stable dissolution and mass transfer [23,24]. This parameter is adopted in this study. Based on the effective machining area, the first-stage current is set to 4 A for 20 s. This stage removes most of the material at a relatively uniform current density, forms relatively straight sidewalls, and suppresses lateral undercutting in the penetrated regions. The current is then increased to 17 A, and a machining time of 8 s is selected through parameter comparison. The short-duration high-current condition provides an electrochemical polishing effect, rapidly removes the remaining unpenetrated regions, and improves the local surface morphology.

After machining, the workpiece is immersed in a low-concentration NaOH solution for 2 min to remove the dry film. It is then rinsed with deionized water and dried. Finally, the temporary connecting structures used for protection during machining and transfer are removed to obtain the monolithic inertial switch for packaging and testing.

**Figure 10:**
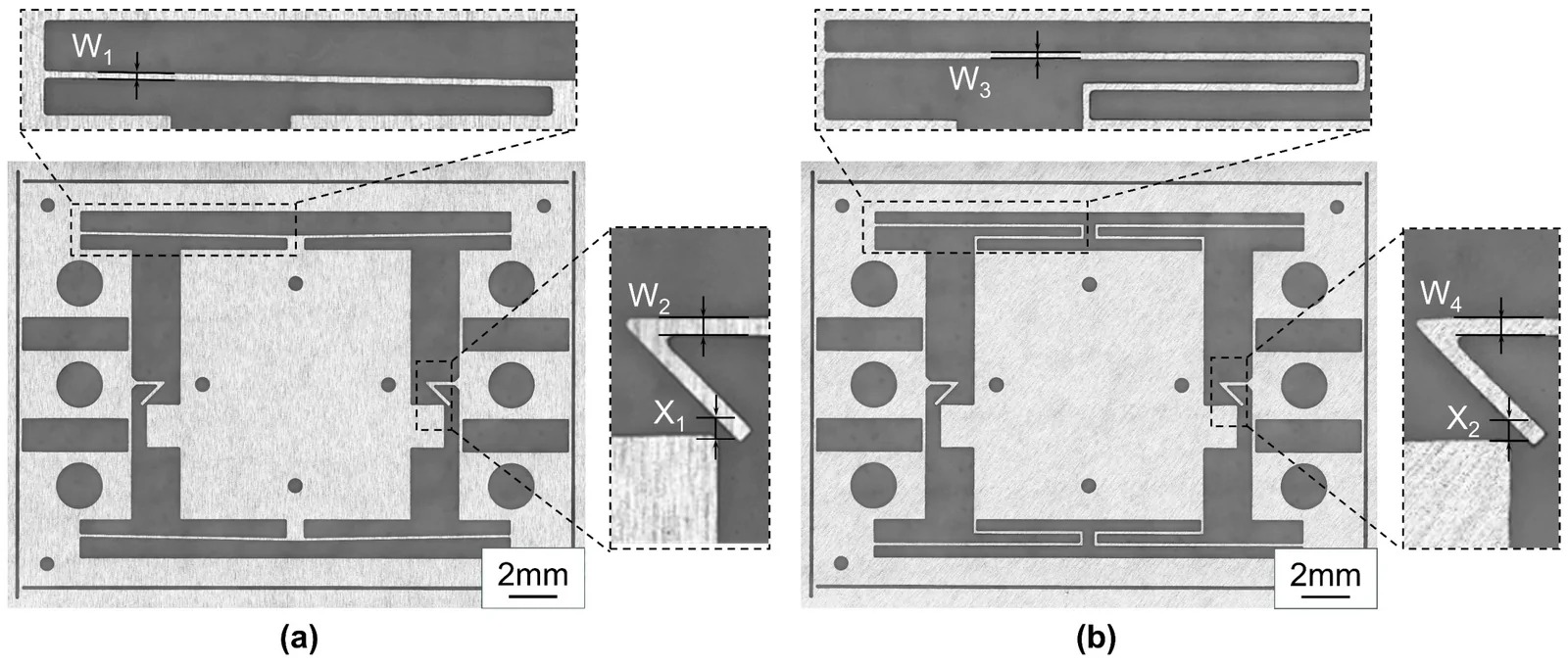
Fabricated inertial-switch specimens and key dimension annotations. (**a**) Nonlinear inertial switch. (**b**) Linear inertial switch.

Multiple nonlinear and linear switches are fabricated to evaluate dimensional consistency and repeatability. The fabricated samples are shown in Figure 10. Both types have complete profiles, with clearly formed spring beams, proof masses, and fixed electrodes. Table 3 lists the mean values of the key dimensions. Their deviations from the design values range from 0.7% to 2.5%. These results demonstrate that IETMEMM enables monolithic fabrication of metal structures with slender beams and narrow gaps.

### 4.2. Testing

**Figure 11:**
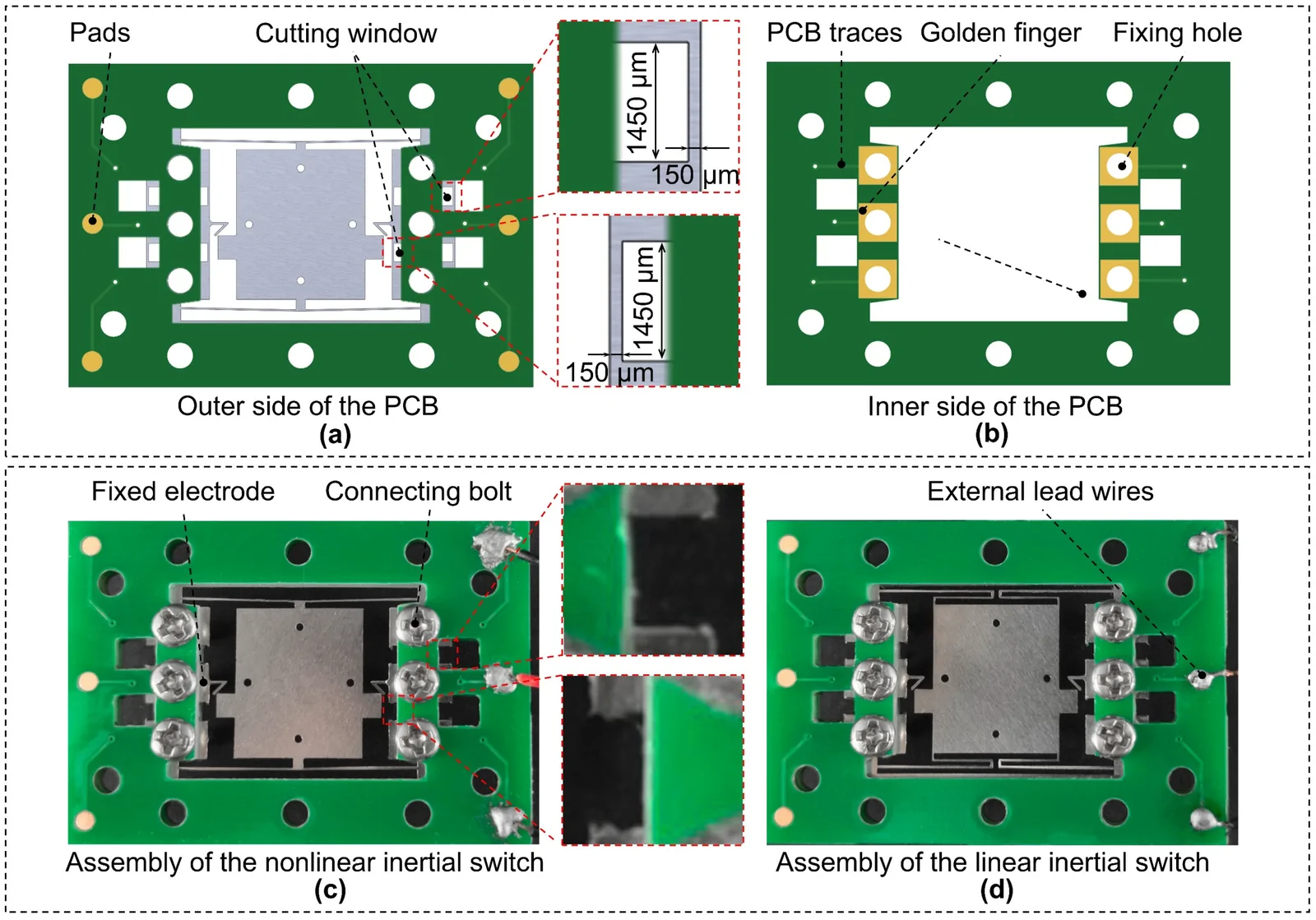
PCB board structure and inertial switch assembly. (**a**) Outer side of the PCB board structure and the local view of cutting windows and the connecting beam before cutting. (**b**) Inner side of the PCB board structure. (**c**) Nonlinear inertial switch assembly and the local view of cutting windows and the connecting beam after cutting. (**d**) Linear inertial switch assembly.

Drop-weight shock tests are performed on the packaged nonlinear and linear inertial switches to verify the improvement in threshold accuracy. A PCB carrier with mounting holes, pads, traces, and cutting windows is designed for device mounting, electrode isolation, and signal extraction, as shown in Figure 11a,b. The mounting holes ensure that the inertial switch and the reference accelerometer receive the same shock input. The pads and traces connect the device to the external detection circuit.

During packaging, the inertial switch is first placed in the reserved PCB window, with the outer frame aligned and attached to the mounting area. The device is then fixed with bolts. To prevent electrode-gap variations caused by twisting or deformation of the electrodes before assembly, the fixed electrodes are temporarily connected to the outer frame by four 150 μm-wide connecting beams, as shown in the enlarged view of Figure 11a. After the device is clamped with the mounting bolts, these temporary connections must be removed to separate the fixed electrodes from the outer frame. A miniature end-cutting plier is used to rapidly cut the connecting beams at their roots through the reserved inner and outer cutting windows. This cutting method produces no residual chips and causes no structural deformation. Finally, the isolated electrodes and common terminal are connected to the signal acquisition circuit by wires. The packaged nonlinear and linear switches are shown in Figure 11c,d. This method enables mechanical mounting and signal extraction without directly soldering the microstructures.

**Figure 12:**
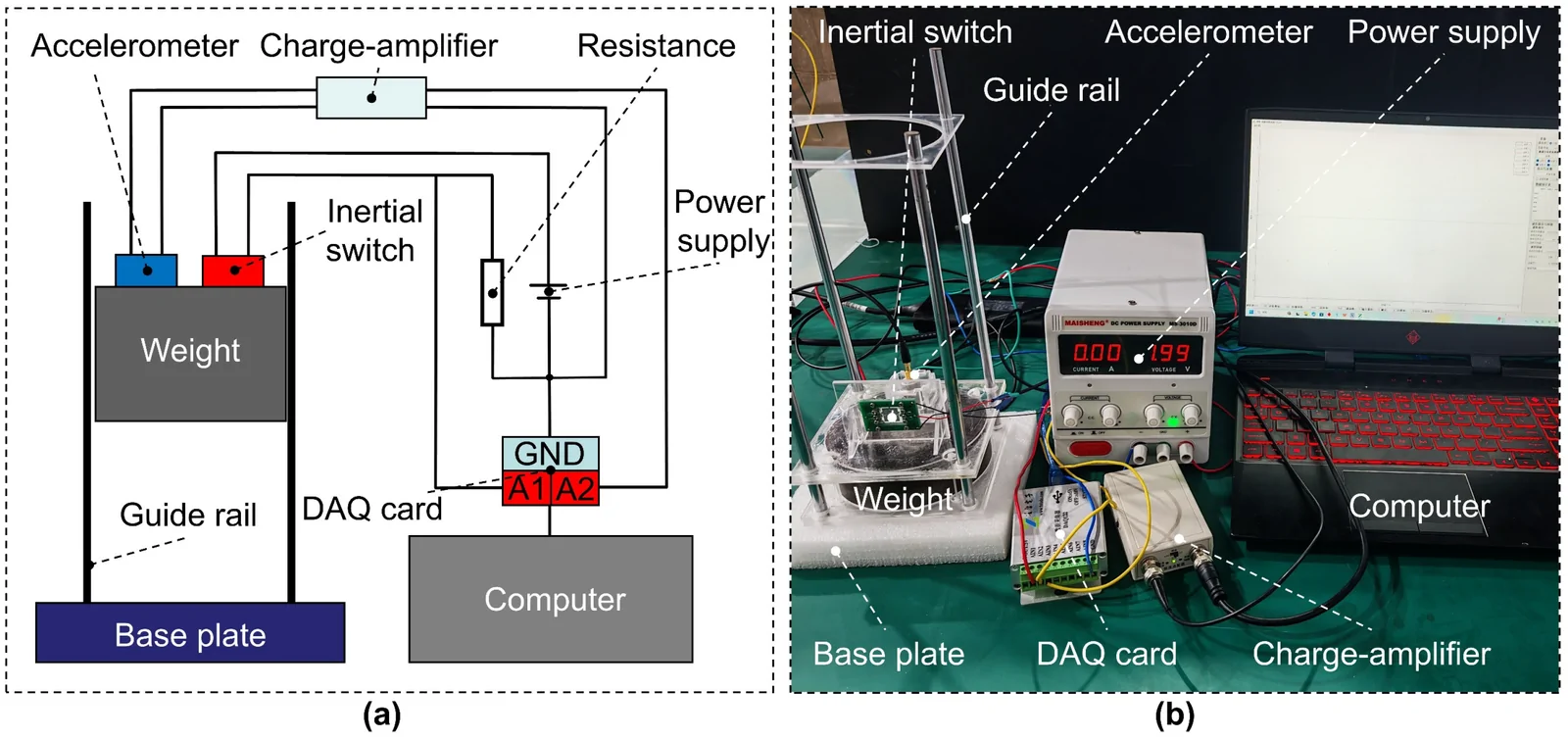
Drop-weight experimental setup. (**a**) Schematic of the testing system. (**b**) Photograph of the testing system.

The schematic and photograph of the experimental setup are shown in Figure 12a,b. The test system consists of a drop-weight impact platform, a reference accelerometer, the inertial switch under test, a signal-conditioning circuit, a data acquisition card, and a computer. During testing, the switch and the reference accelerometer are mounted together on the drop mass. The sensing direction of the switch is aligned with the shock direction so that both devices receive the same shock input.

The drop mass falls freely from a preset height and strikes a cushioning plate, producing an approximately half-sine acceleration pulse. The peak acceleration is adjusted by changing the drop height, while the pulse width is controlled by using cushioning plates with different stiffnesses or thicknesses. The reference accelerometer measures the peak acceleration and pulse width in real time. The acceleration signal and switch output are acquired synchronously and recorded by the computer. A clear voltage jump indicates electrical contact between the proof mass and the fixed electrode and is therefore taken as the triggering event.

Approximate half-sine shocks with pulse widths of 4, 6, 8, and 10 ms are used. The peak acceleration is varied by gradually adjusting the drop height. The black curve represents the acceleration response signal (ARS), and the red curve represents the switch closure signal (SCS). A transient SCS pulse indicates effective switch closure.

Repeated tests are performed at each pulse width and acceleration level for two samples of each switch, to reduce the effects of accidental contact, signal fluctuation, and single-impact error. The results are listed in Table 4 and Table 5, respectively. Acceleration values are expressed in units of gravitational acceleration (g). The switch is considered stably closed when repeated tests at similar peak accelerations consistently produce a stable SCS, and this state is denoted as 1. It is considered stably open when no valid SCS is observed, and this state is denoted as 0. For each device, six tests are performed under each condition. The interval between adjacent stable-open and stable-closed conditions is defined as the triggering transition range. Its midpoint is taken as the measured dynamic triggering threshold. This experimental criterion corresponds to the simulated triggering condition in which the proof-mass displacement reaches the 180 μm electrode gap. For the nonlinear switch, the mean absolute difference between the two samples ranges from 0.07 to 0.93 g, corresponding to relative differences of 0.25–3.36%, with an average of 1.53%. For the linear switch, the corresponding values are 0.12–0.58 g, 0.76–3.83%, and 1.76%, respectively. Overall, both switches show good test repeatability and sample-to-sample consistency, with no obvious systematic deviation.

**Figure 13:**
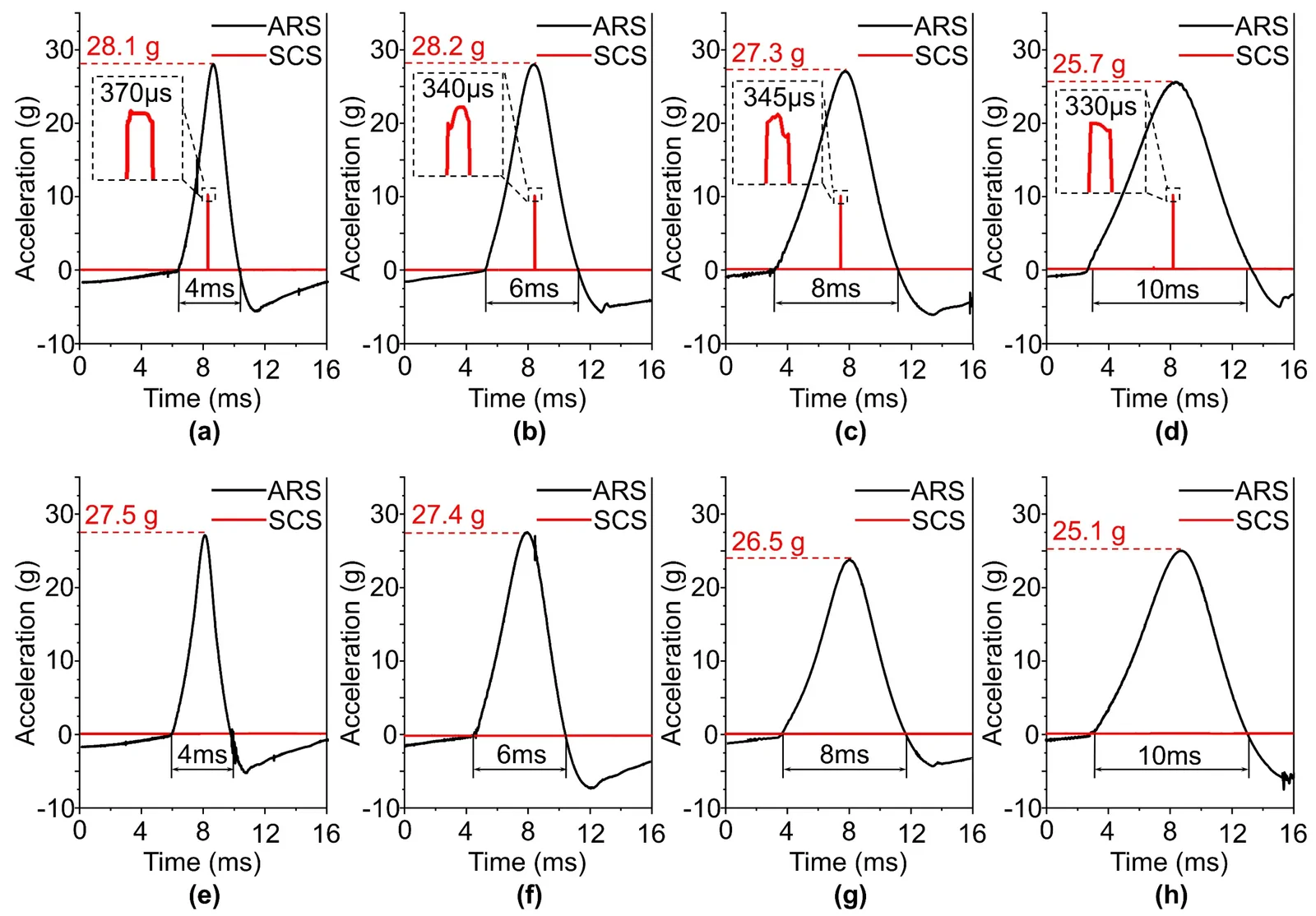
Threshold–test results of the nonlinear inertial switch at different pulse widths. The black curve represents the acceleration response signal (ARS), and the red curve represents the switch closure signal (SCS). (**a**–**d**) Stable–closure boundary responses. (**e**–**h**) Stable–open boundary responses.

**Figure 14:**
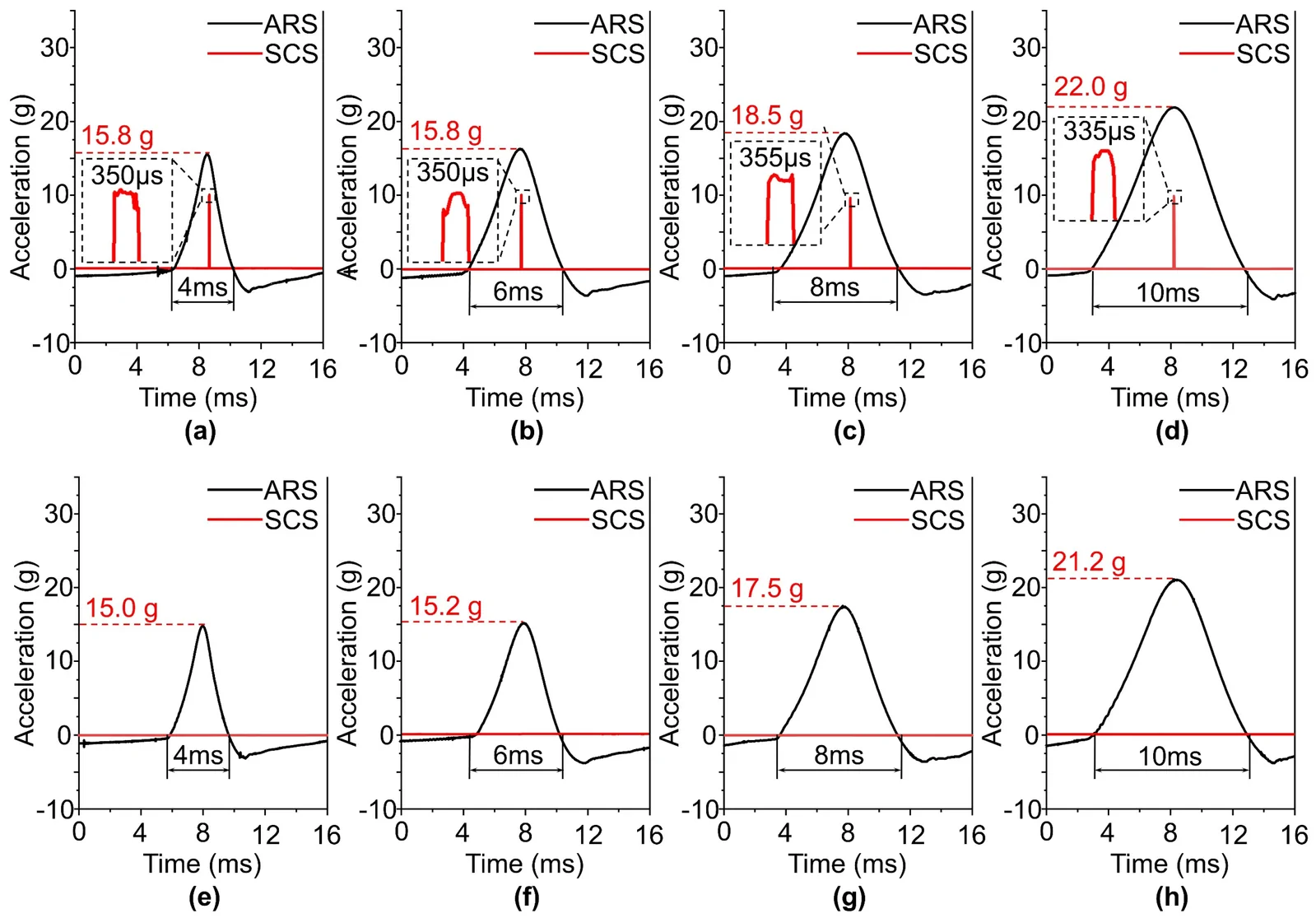
Threshold–test results of the linear inertial switch at different pulse widths. The black curve represents the acceleration response signal (ARS), and the red curve represents the switch closure signal (SCS). (**a**–**d**) Stable–closure boundary responses. (**e**–**h**) Stable–open boundary responses.

Accordingly, the triggering boundary is determined from the maximum stable-open value and the minimum stable-closed value listed in the tables. Figure 13 shows the critical triggering responses of the nonlinear inertial switch at different pulse widths. At 4 ms, the switch remains stably open at 27.5 g and closes stably at 28.1 g. The estimated dynamic triggering threshold is therefore 27.8 g. Using the same method, the thresholds at 6, 8, and 10 ms are 27.8, 26.9, and 25.4 g, respectively. All values remain close to the quasi-static design threshold of 27.5 g. Figure 14 shows the critical triggering responses of the linear inertial switch. Its dynamic triggering thresholds at 4, 6, 8, and 10 ms are 15.4, 15.5, 18.0, and 21.6 g, respectively. These values are all markedly below the design threshold and vary strongly with pulse width.

**Figure 15:**
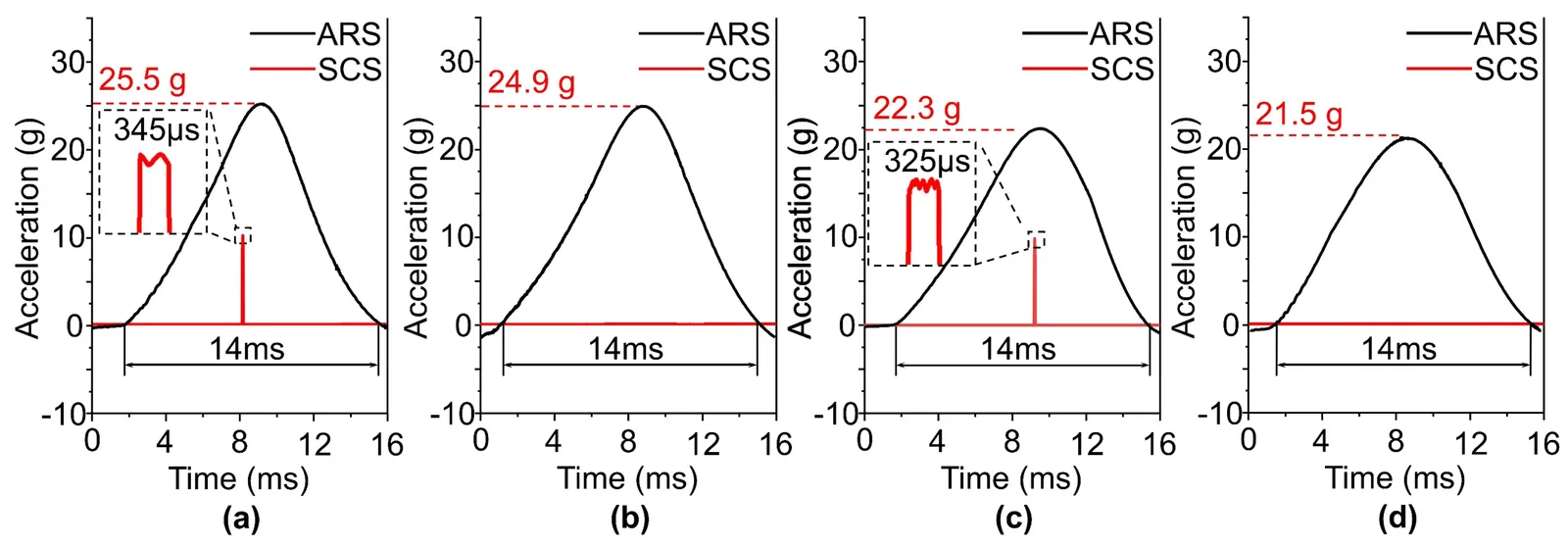
Threshold–test results of the inertial switches at a pulse width of 14 ms. The black curve represents the acceleration response signal (ARS), and the red curve represents the switch closure signal (SCS). (**a**,**b**) Nonlinear inertial switch. (**c**,**d**) Linear inertial switch.

As shown in Figure 13 and Figure 14, the measured thresholds of the nonlinear switch range from 25.4 to 27.8 g, with a span of 2.4 g. At 4 and 6 ms, the threshold differs from the design value by only 0.3 g. In contrast, the thresholds of the linear switch range from 15.4 to 21.6 g, with a span of 6.2 g, and shift toward lower accelerations. The experimental trends agree with the dynamic simulation results. The nonlinear structure concentrates the triggering boundary near the design threshold, thereby reducing dynamic threshold deviation and improving measured threshold accuracy. Figure 15a–d show the test results of the nonlinear and linear switches at a pulse width of 14 ms, respectively. Their dynamic triggering thresholds are 25.2 and 21.9 g, respectively. These values are close to those obtained at 10 ms and are consistent with the simulation trends, indicating that the switch response approaches the quasi-static regime when the pulse width exceeds 10 ms.

The experimental results of both devices agree with the transient dynamic simulations. The open-to-closed transition boundary of the nonlinear switch remains close to the design threshold, whereas the triggering boundary of the linear switch shifts markedly toward lower accelerations. This result validates the proposed nonlinear threshold-regulation mechanism.

**Figure 16:**
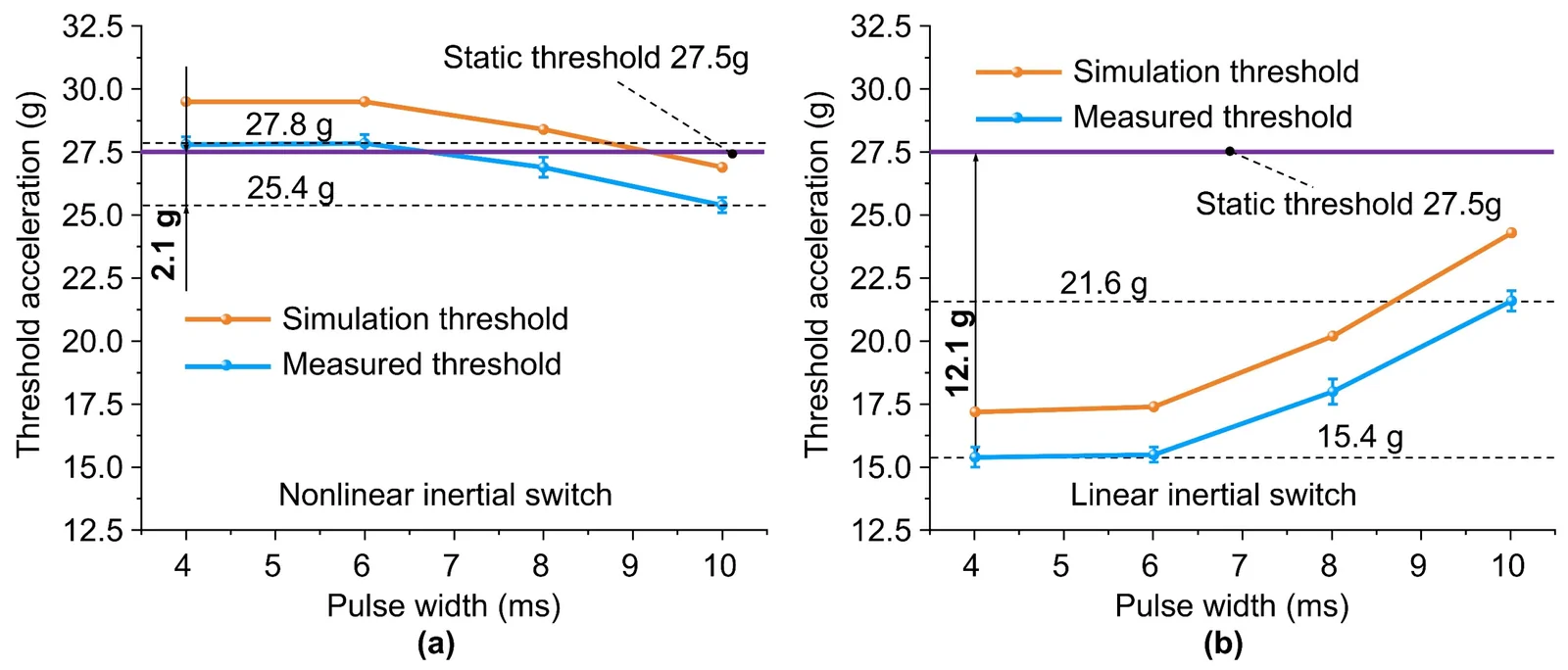
Comparison of the simulated and measured triggering thresholds at different pulse widths. The measured threshold was defined as the midpoint between the maximum stable-open acceleration and the minimum stable-closure acceleration. The error bars indicate the corresponding triggering transition ranges. (**a**) Nonlinear inertial switch. (**b**) Linear inertial switch.

Figure 16 compares the simulated and measured dynamic triggering thresholds of the two inertial switches at different shock pulse widths. For the nonlinear switch, the simulated thresholds at 4, 6, 8, and 10 ms are 29.5, 29.5, 28.4, and 26.9 g, respectively, while the measured thresholds are 27.8, 27.8, 26.9, and 25.4 g. The measured values are 1.5–1.7 g lower than the simulated values, with a mean difference of 1.60 g. For the linear switch, the simulated thresholds are 17.2, 17.4, 20.2, and 24.3 g, and the measured thresholds are 15.4, 15.5, 18.0, and 21.6 g, with a mean difference of 2.15 g. Although the measured thresholds are generally lower than the simulated values, both switches exhibit pulse-width-dependent trends consistent with the simulations, indicating that the dynamic model reasonably describes their triggering behavior. The simulation–experiment differences may arise from dimensional deviations, packaging-induced boundary variations, deviations of the actual shock waveform from an ideal half-sine pulse, and errors in signal acquisition and threshold determination. Fabrication errors may be the main source: a reduced beam width decreases spring stiffness, while a reduced electrode gap shortens the triggering displacement, both leading to lower measured thresholds.

With the quasi-static design threshold of 27.5 g as the reference, the measured dynamic triggering thresholds of the nonlinear switch range from 25.4 to 27.8 g over pulse widths of 4–10 ms, with a threshold span of only 2.4 g. The absolute and relative deviations range from 0.3 to 2.1 g and from 1.1% to 7.6%, respectively, and the overall threshold accuracy reaches 92.4%. In contrast, the measured thresholds of the linear switch range from 15.4 to 21.6 g, with a span of 6.2 g. Its absolute and relative deviations range from 5.9 to 12.1 g and from 21.5% to 44.0%, respectively, and the overall threshold accuracy is only 56.0%. These results show that the nonlinear switch has a more concentrated threshold distribution and better maintains the quasi-static design threshold under different pulse widths.

Compared with the linear reference switch, the nonlinear switch reduces the mean simulation–experiment difference from 2.15 to 1.60 g. The threshold span decreases from 6.2 to 2.4 g. The maximum absolute deviation decreases from 12.1 to 2.1 g, while the mean absolute deviation decreases from 9.88 to 0.83 g. The overall threshold accuracy increases from 56.0% to 92.4%. These results are consistent with the theoretical analysis, quasi-static simulations, and transient dynamic simulations. The proposed geometric nonlinearity concentrates high displacement sensitivity near the design threshold, thereby improving threshold discrimination and suppressing dynamic threshold drift caused by pulse-width variation. The experiments therefore validate the proposed nonlinear threshold-regulation method and demonstrate its ability to significantly improve the dynamic threshold accuracy and stability of metal MEMS inertial switches.

## 5. Conclusions

To improve the threshold accuracy of MEMS inertial switches, this study proposes a monolithic metal nonlinear MEMS inertial switch fabricated by IETMEMM. The device is validated through finite element simulations, fabrication, and drop-weight shock tests. The main conclusions are as follows:

The proof mass is supported by two sets of inclined beams with asymmetric initial angles. Geometric nonlinearity makes the stiffness vary with displacement and concentrates high displacement sensitivity near the design threshold. This response improves threshold discrimination and reduces threshold deviation.

The nonlinear and linear reference switches both have a quasi-static design threshold of 27.5 g. In the dynamic simulations, the nonlinear switch achieves an overall threshold accuracy of 92.7% over pulse widths of 4–10 ms, compared with 62.5% for the linear switch.

The proof mass, suspension springs, fixed electrodes, and outer frame are monolithically fabricated from a 50 μm-thick 304 stainless-steel sheet by IETMEMM. The key dimensional deviations range from 0.7% to 2.5%, confirming the feasibility of fabricating slender beams, narrow gaps, and complex metal structures.

The measured thresholds of the nonlinear switch at pulse widths of 4, 6, 8, and 10 ms are 27.8, 27.8, 26.9, and 25.4 g, respectively. Its overall threshold accuracy reaches 92.4%. Compared with the linear switch, the threshold span is reduced by 61.3%, and the overall threshold accuracy is improved by 36.4 percentage points. These results validate the proposed nonlinear threshold-regulation mechanism.

Advantages and Limitations. The triggering thresholds of the switches were tested over a pulse-width range of 4–14 ms, and the switches exhibited high threshold accuracy. These results indicate that the proposed nonlinear threshold-regulation strategy provides a practical approach for improving the reliability and consistency of metal MEMS inertial switches, particularly in applications requiring accurate shock threshold detection. However, due to limitations in the current testing conditions, the effects of different shock waveforms, temperature variations, off-axis acceleration, repeated cycling/fatigue, and overload conditions remain to be further investigated.

## Figures and Tables

**Table 1:** Main geometric parameters of the two inertial switches.

Component	Geometric Parameter	Value
Nonlinear spring	*S*_1_	9000 μm
	*φ*_1_	1.001°
	*φ*_2_	1.146°
	*W*_1_	120 μm
Linear spring	*S*_2_	9130 μm
	*h*_1_	400 μm
	*h*_2_	500 μm
Fixed electrode	*X*	180 μm
	*W*_2_	150 μm
	*φ*_3_	45°
Proof mass	*D*_1_	10,050 μm
	*H*_1_	11,750 μm
Frame	*D*_2_	24,110 μm
	*H*_2_	17,590 μm

**Table 2:** Simulated triggering thresholds of the inertial switches.

Pulse Width (ms)	Nonlinear-Switch Threshold (g)	Linear-Switch Threshold (g)
4	29.5	17.2
6	29.5	17.4
8	28.4	20.2
10	26.9	24.3
14	26.8	24.4
Static	27.5	27.5

**Table 3:** Measured values of key geometric parameters.

**Device Structure**	**Geometric Parameter**	**Measured Value (μm)**
Nonlinear inertial switch	*W*_1_	118
	*W*_2_	152
	*X*_1_	177
Linear inertial switch	*W*_3_	117
	*W*_4_	151
	*X*_2_	178

**Table 4:** Nonlinear inertial switch testing and statistics.

Testing and Statistics	4 ms	6 ms	8 ms	10 ms	14 ms
Device 1, Test 1	28.3 (1)	26.8 (0)	25.7 (0)	26.3 (1)	24.9 (0)
Device 1, Test 2	27.1 (0)	26.6 (0)	27.7 (1)	25.1 (0)	24.5 (0)
Device 1, Test 3	28.3 (1)	28.4 (1)	26.5 (0)	25.9 (1)	25.7 (1)
Device 1, Test 4	27.3 (0)	26.9 (0)	26.4 (0)	24.3 (0)	24.6 (0)
Device 1, Test 5	28.9 (1)	27.1 (0)	27.6 (1)	25.0 (0)	25.5 (1)
Device 1, Test 6	28.5 (1)	28.2 (1)	27.4 (1)	24.8 (0)	26.0 (1)
Device 2, Test 1	27.5 (0)	28.4 (1)	27.4 (1)	24.7 (0)	25.7 (1)
Device 2, Test 2	28.6 (1)	29.0 (1)	27.3 (1)	24.3 (0)	24.5 (0)
Device 2, Test 3	26.6 (0)	28.9 (1)	26.1 (0)	26.1 (1)	24.2 (0)
Device 2, Test 4	26.9 (0)	27.1 (0)	26.4 (0)	25.7 (1)	25.7 (1)
Device 2, Test 5	26.9 (0)	27.4 (0)	28.1 (1)	26.1 (1)	25.7 (1)
Device 2, Test 6	28.1 (1)	28.8 (1)	26.4 (0)	26.4 (1)	24.6 (0)
Overall Mean (*n* = 12)	27.750	27.800	26.917	25.392	25.133
Standard Deviation (SD)	0.786	0.901	0.752	0.777	0.637
95% CI, Lower Bound	27.251	27.228	26.439	24.898	24.728
95% CI, Upper Bound	28.249	28.372	27.394	25.886	25.538
Device 1 Mean	28.067	27.333	26.883	25.233	25.200
Device 2 Mean	27.433	28.267	26.950	25.550	25.067
Absolute Inter-Device Mean Difference (|D1 – D2|)	0.633	0.933	0.067	0.317	0.133

**Table 5:** Linear inertial switch testing and statistics.

Testing and Statistics	4 ms	6 ms	8 ms	10 ms	14 ms
Device 1, Test 1	14.4 (0)	14.6 (0)	17.1 (0)	21.2 (0)	20.8 (0)
Device 1, Test 2	14.2 (0)	16.1 (1)	19.2 (1)	22.2 (1)	22.4 (1)
Device 1, Test 3	16.1 (1)	16.0 (1)	17.5 (0)	20.3 (0)	22.5 (1)
Device 1, Test 4	15.0 (0)	14.5 (0)	19.1 (1)	22.7 (1)	22.6 (1)
Device 1, Test 5	15.8 (1)	16.6 (1)	17.3 (0)	21.0 (0)	20.8 (0)
Device 1, Test 6	14.2 (0)	14.4 (0)	18.5 (1)	20.7 (0)	23.0 (1)
Device 2, Test 1	16.3 (1)	14.9 (0)	18.7 (1)	21.0 (0)	22.3 (1)
Device 2, Test 2	16.0 (1)	15.8 (1)	19.1 (1)	20.3 (0)	20.9 (0)
Device 2, Test 3	14.6 (0)	15.2 (0)	17.2 (0)	22.5 (1)	22.6 (1)
Device 2, Test 4	15.9 (1)	15.9 (1)	19.1 (1)	22.1 (1)	21.2 (0)
Device 2, Test 5	14.3 (0)	16.5 (1)	16.7 (0)	22.0 (1)	21.3 (0)
Device 2, Test 6	16.1 (1)	14.6 (0)	16.9 (0)	22.2 (1)	21.5 (0)
Overall Mean (*n* = 12)	15.242	15.425	18.033	21.517	21.825
Standard Deviation (SD)	0.861	0.814	0.995	0.860	0.816
95% CI, Lower Bound	14.695	14.908	17.401	20.970	21.307
95% CI, Upper Bound	15.789	15.942	18.665	22.063	22.343
Device 1 Mean	14.950	15.367	18.117	21.350	22.017
Device 2 Mean	15.533	15.483	17.950	21.683	21.633
Absolute Inter-Device Mean Difference (|D1 – D2|)	0.583	0.117	0.167	0.333	0.383

Note: Each entry is expressed as *a* (state), where *a* is the measured peak acceleration in g, and state = 1 and 0 indicate stable closure and stable open, respectively.

## Data Availability

The raw data supporting the conclusions of this article will be made available by the authors on request.

## Abbreviations

The following abbreviations are used in this manuscript:

MEMS	Microelectromechanical systems
IETMEMM	Induction-electrode through-mask electrochemical micromachining
TMEMM	Through-mask electrochemical micromachining
PCB	Printed circuit board
ARS	Acceleration response signal
SCS	Switch closure signal
